# The impact of mobile health interventions on maternal-neonatal outcomes in women with gestational diabetes mellitus: a systematic review and meta-analysis

**DOI:** 10.3389/fendo.2025.1707520

**Published:** 2025-12-19

**Authors:** Qiaofang Yang, Yali Yang, Guilan Nie, Jianyi Lou

**Affiliations:** 1 Department of Obstetrics, Jinhua Maternal and Child Health Care Hospital, Jinhua, Zhejiang, China; 2 Department of Obstetrics, Affiliated Jinhua Hospital, Zhejiang University School of Medicine, Jinhua, Zhejiang, China

**Keywords:** gestational diabetes mellitus, maternal and neonatal outcomes, mobile health interventions, systematic review, meta-analysis

## Abstract

**Background:**

Gestational diabetes mellitus (GDM), a prevalent complication during pregnancy, is closely associated with an elevated risk of adverse maternal and neonatal outcomes. Mobile health (mHealth) technologies have emerged as convenient tools for GDM management; however, their clinical efficacy in improving maternal and neonatal outcomes among GDM-affected pregnant individuals remains to be comprehensively evaluated.

**Objective:**

The study aims to evaluate the effectiveness of mHealth interventions in improving maternal and neonatal outcomes among pregnant individuals with GDM.

**Methods:**

This study systematically searched the PubMed, Web of Science, Scopus, Cochrane Library, and EMBASE databases from their inception to July 23, 2025. Two researchers independently screened the studies, extracted data, and assessed quality. All data analyses were performed using STATA 17.0 software.

**Results:**

Compared with routine care, mHealth interventions significantly reduced the risk of cesarean section (OR 0.76, 95% CI 0.63–0.91) and emergency cesarean (OR 0.55, 95% CI 0.39–0.77) among women with GDM. Additionally, a significant reduction in the risk of composite neonatal complications was observed (OR 0.63, 95% CI 0.44–0.89). Furthermore, mHealth interventions significantly improved 2-hour postprandial blood glucose levels (SMD −0.36, 95% CI −0.53 to −0.19). A trend toward reduced gestational weight gain was also noted in the mHealth intervention group (SMD −0.37, 95% CI −0.83 to 0.08).

**Conclusion:**

mHealth interventions can reduce the risk of cesarean section rate and emergency cesarean section, as well as the risk of composite neonatal complications. mHealth interventions may also improve two-hour postprandial blood glucose control in pregnant women with GDM and can effectively supplement conventional clinical care for GDM.

**Systematic Review Registration:**

https://www.crd.york.ac.uk/prospero/, identifier CRD420251149505.

## 1 Introduction

Gestational Diabetes Mellitus (GDM) is a common metabolic disorder during pregnancy, defined as diabetes that is first diagnosed or occurs during gestation, excluding women with pre-existing diabetes mellitus (1). Its core pathological mechanism involves increased insulin resistance and inadequate compensatory function of pancreatic β-cells, which is closely associated with hormonal changes and metabolic dysregulation during pregnancy (2). Multiple studies have indicated that the global prevalence of GDM is increasing annually, influenced by rising obesity rates, lifestyle factors, and unhealthy dietary habits (3, 4). According to statistics, the global prevalence rate in 2021 was 13.4% (5) and continues to rise. GDM not only adversely affects maternal health (1) but also poses risks to the newborn, including macrosomia, dystocia, neonatal hypoglycemia, and respiratory distress syndrome, among others (6). Although GDM typically manifests transiently during the perinatal period and often resolves postpartum, its potential complications extend well beyond the gestational period. Studies have shown that women with a history of GDM have a significantly increased risk of recurrence in subsequent pregnancies (7). Furthermore, GDM has been identified as an independent risk factor for various chronic conditions, including cardiovascular disease and type 2 diabetes mellitus (8, 9). These adverse effects include short-term complications during gestation and the perinatal period. However, they may extend for decades postpartum, leading to long-term detrimental impacts on both maternal and infant health (10, 11). Concurrently, the associated medical and nursing costs and the consumption of social resources impose a substantial economic burden on families and public health systems. Therefore, it is essential to strengthen early screening, standardized management, and long-term follow-up interventions for GDM, which may help mitigate related health risks and improve maternal and infant outcomes.

However, the traditional GDM management model has the following limitations. In terms of blood glucose monitoring, discrete sampling data fail to adequately capture dynamic fluctuations, resulting in reduced timeliness of interventions (12, 13). Regarding patient compliance, self-management based on paper records is significantly influenced by educational level and cognitive differences, making it difficult to maintain adherence (14, 15). In the allocation of medical resources, resource-limited areas face constraints in human and equipment capacity, hindering comprehensive management of high-risk populations; moreover, standardized protocols are unable to accommodate the clinical heterogeneity of GDM (16). Regarding behavioral interventions, static educational approaches (e.g., printed manuals) have limited effectiveness in promoting long-term self-management behaviors (17). Therefore, these limitations highlight the need for innovative healthcare models in the management of GDM.

The rapid advancement of Mobile Health (mHealth) technology offers the potential to overcome the limitations of traditional interventions. The core of mHealth lies in updating healthcare service models through mobile devices (e.g., smartphones, wearable sensors), remote monitoring platforms, and artificial intelligence algorithms (18). This facilitates a shift in monitoring patterns from “intermittent” to “continuous.” By integrating wearable glucose monitoring devices with mHealth, key data such as postprandial glucose peaks and nocturnal glucose fluctuations can be captured in real-time, enabling healthcare providers to accurately assess patients’ metabolic status and provide a scientific basis for adjusting intervention strategies (19, 20). The patient-provider interaction model is transformed from “passive follow-up” to “active management.” mHealth platforms facilitate real-time communication that transcends temporal and spatial constraints, allowing pregnant women to report symptoms and seek advice promptly. At the same time, healthcare providers deliver proactive interventions through intelligent reminders and personalized guidance, thereby enhancing patient adherence (21, 22). Furthermore, the intervention approach evolves from “standardized” to “personalized.” Utilizing recommendation algorithms based on big data analysis, and incorporating individual characteristics such as blood glucose levels, body weight, and dietary and exercise habits, enables the dynamic optimization of dietary plans, physical activity, and insulin regimens to accommodate the heterogeneous management needs of GDM (23, 24). Numerous studies have demonstrated that mHealth interventions can enhance and optimize patient-provider communication, improve healthcare accessibility, reduce medical costs, rationalize the allocation of medical resources, strengthen patients’ self-management capabilities, and promote the realization of personalized medicine and long-term management (25, 26).

In recent years, mHealth has garnered significant attention in healthcare and has been widely integrated into health systems to support self-symptom management for conditions such as cancer and arthritis (27, 28). However, the conclusions of existing studies regarding the effectiveness of mHealth interventions on maternal and infant outcomes in women with GDM remain inconsistent. Some studies have demonstrated that mHealth interventions significantly reduce the risk of emergency cesarean section and composite neonatal complications (29, 30). In contrast, others have found no association between mHealth interventions and these outcomes (31, 32). Furthermore, while certain studies have confirmed that mHealth interventions effectively improve 2-hour postprandial blood glucose levels and mitigate gestational weight gain in women with GDM (32, 33), others have reported inconsistent or non-significant results (34, 35). Therefore, given that cesarean section, emergency cesarean section, 2-hour postprandial blood glucose, gestational weight gain in pregnant women with GDM, and composite neonatal complications in their offspring are all core indicators for perinatal quality assessment. Considering the substantial controversy in existing research findings, we conducted a meta-analysis to comprehensively and systematically evaluate the efficacy of mHealth interventions in pregnant women with GDM and their offspring.

## 2 Methods

The study was reported in accordance with the Preferred Reporting Items for Systematic Reviews and Meta-Analyses (PRISMA) (36). This systematic review and meta-analysis were registered in PROSPERO (CRD420251149505).

### 2.1 Search strategy

A comprehensive electronic literature search was conducted across PubMed, Web of Science, Scopus, Cochrane Library, and EMBASE from the inception of each database up to July 23, 2025. The search strategy incorporated both MeSH and free-text terms. The search strategy for PubMed is shown in **Supplementary Material Table S1**. It was adapted for the other databases. Search syntax was adapted according to the specific indexing systems and query rules of each database. A manual search of reference lists from relevant articles was also performed to identify additional eligible studies.

### 2.2 Eligibility criteria

Literature retrieval was limited to published English-language articles. Eligible studies were required to meet the following criteria:

#### 2.2.1 Study population

The target population consisted of pregnant women with GDM.

#### 2.2.2 Study design

Randomized controlled trials (RCTs).

#### 2.2.3 Intervention

The intervention group received mHealth interventions delivered via mobile devices (e.g., smartphones) and digital technologies (e.g., mobile apps, wearable sensors, website).

#### 2.2.4 Control

The control group was subjected to conventional care interventions, health education, usual care, or other non-mHealth intervention measures.

#### 2.2.5 Outcome measures

At least one of the following outcome indicators was required: cesarean section, emergency cesarean section, composite neonatal complications, 2-hour blood glucose level, and gestational weight gain.

#### 2.2.6 Data characteristics

Mean and standard deviation.

Studies meeting the above criteria were included.

### 2.3 Study selection

All retrieved records from the databases were imported into EndNote X9 software for deduplication and literature management. Two independent reviewers (QFY and YLY) initially screened titles and abstracts based on predefined inclusion criteria. For studies that preliminarily met the criteria, full texts were retrieved and further screened to determine final inclusion. Any disagreements between the two reviewers during the screening process were resolved through discussion; when necessary, a third reviewer (JYL) was consulted to provide arbitration.

### 2.4 Data extraction

This study strictly adhered to the PRISMA statement for data extraction to ensure methodological systematicity. Two reviewers (QFY and YLY) independently extracted data using a pre-tested data extraction form, and a third author (JYL) cross-verified the accuracy of the results. The extracted data included publication details (authors, year of study, country), sample size, intervention methods for both the intervention and control groups, duration of intervention, and primary outcomes.

### 2.5 Quality assessment

Two authors (QFY and YLY) independently assessed the risk of bias, methodological quality, and certainty of evidence for the included studies. Any assessment discrepancies were resolved through discussion with a third reviewer (JYL) until consensus was reached. The risk of bias was evaluated using the revised Cochrane Risk of Bias tool (RoB 2), focusing on the following five domains: the randomization process, deviations from intended interventions, missing outcome data, outcome measurement, and selection of the reported result (37).

### 2.6 Data synthesis and analysis

Given the anticipated heterogeneity among the included studies, a random-effects model was employed to synthesize the pooled estimates of post-intervention effects. Standardized mean differences (SMDs) with 95% confidence intervals (CIs) were calculated using the inverse variance method for continuous variables. Following Cohen (38), effect sizes were interpreted as small (0.2), moderate (0.5), or large (0.8). The degree of heterogeneity was assessed using the I² statistic, with the following thresholds: I² < 25% indicating low heterogeneity, 25% ≤ I² < 50% moderate heterogeneity, 50% ≤ I² < 75% high heterogeneity, and I² ≥ 75% indicating very high heterogeneity (39). Publication bias was evaluated through visual inspection of funnel plot symmetry and by calculating Begg’s and Egger’s test values (40, 41). Sensitivity analysis was conducted using the leave-one-out method to assess the robustness of the pooled results (42). All statistical analyses were performed using STATA 17.0.

## 3 Results

### 3.1 Compliance with the registered protocol

There were no other inconsistencies with the pre-registration protocol.

### 3.2 Study selection

**Figure 1:**
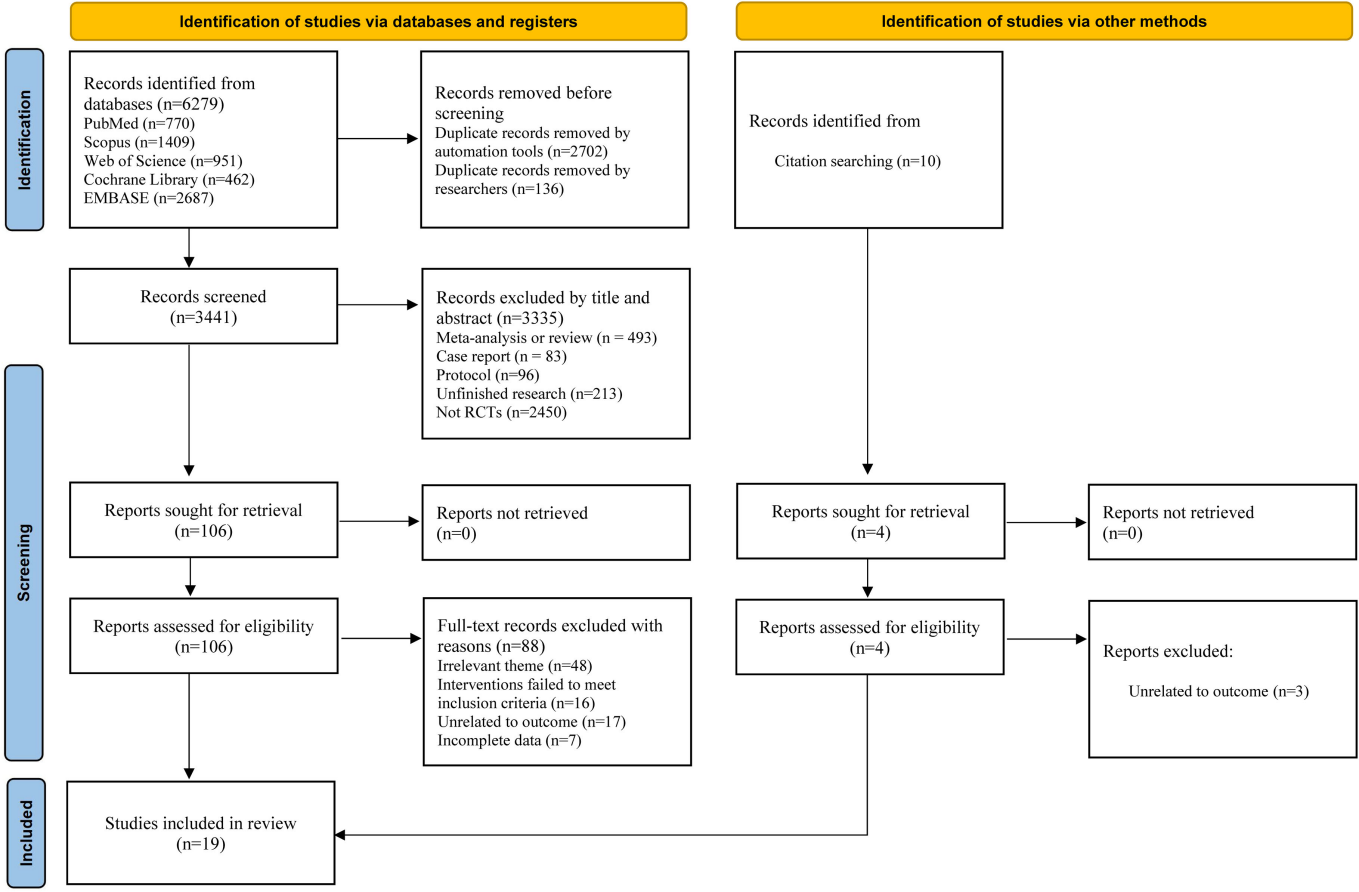
PRISMA flow chart for study selection.

The study selection process and reasons for exclusion are illustrated in **Figure 1**. A total of 6,279 records were retrieved from five databases. After removing duplicates, 3,441 potentially eligible studies were identified. Based on title and abstract screening, 106 studies were initially included. After full-text retrieval and evaluation, 18 RCTs met the inclusion criteria. Among the 88 studies, 44 were excluded due to irrelevant research topics, 16 due to mismatched intervention methods, 17 due to the absence of relevant outcome measures, and 7 due to incomplete data. Additionally, 10 studies were identified through tracking and supplementary searches of relevant citations. After applying the inclusion and exclusion criteria, one more study was included. Ultimately, 19 studies were included in the meta-analysis (29–35, 43–54).

### 3.3 Study characteristics

This meta-analysis included 19 randomized controlled trials conducted between 2007 and 2023, which investigated the effects of mHealth interventions on maternal and neonatal outcomes in pregnant women with GDM. The included studies were conducted across multiple countries and regions, including Asia (China), North America (the United States, Canada), and Europe (Spain, the United Kingdom). 1821 pregnant women with GDM were enrolled across these studies, with sample sizes ranging from 21 to 240 per study. All 19 included studies utilized usual care as the control, while the experimental groups received remote interventions based on mHealth tools such as smartphones and applications. The intervention groups involved diverse types of applications, including mobile phones, the Internet, smartphones (e.g., WeChat), web-based telemedicine systems (e.g., DiabeTIC website), and telehomecare (THCa) systems. Regarding intervention duration, 15 studies continued the intervention until delivery, one study lasted 6 months, one for 3.5 months (14 weeks), and two did not report the duration. The primary outcome measures in this analysis included cesarean section, composite neonatal complications, 2-hour oral glucose tolerance test, and gestational weight gain, among others. More detailed information regarding the main results of each study is presented in **Table 1**.

**Table 1:** Characteristics of the included studies.

Author, year	Country	Sample		Intervention methods		Duration of intervention	Main outcomes
		Intervention group	Control group	Intervention group	Control group		
Borgen et al., 2019 (29)	Norway	115	123	Mobile phone	Usual care	To birth	Cesarean section, Emergency cesarean
Carral et al., 2015 (46)	Spain	40	64	Web-based telemedicine system(DiabeTIC website)	Usual care	To birth	Cesarean section, Composite neonatal complication, Gestational weight gain
Durnwald et al., 2016 (35)	United States	49	52	Telephone	Usual care	To birth	Cesarean section, h-OGTT, Gestational weight gain
Given et al., 2015 (47)	United Kingdom	24	26	Internet	Usual care	To birth	Cesarean section
Guo et al., 2019 (33)	China	64	60	Mobile phone	Usual care	To birth	Cesarean section, h-OGTT, Gestational weight gain
Homko et al., 2007 (43)	United States	32	25	Internet	Usual care	To birth	Composite neonatal complication, 2-h-OGTT
Homko et al., 2012 (45)	United States	40	40	Internet	Usual care	To birth	Cesarean section, 2-h-OGTT
Lemelin et al., 2020 (51)	Canada	80	81	Telehomecare (THCa) system	Usual care	Not Available	Cesarean section, Emergency cesarean
Mackillop et al., 2018 (48)	United Kingdom	101	102	Mobile phone	Usual care	To birth	Cesarean section, Emergency cesarean, Gestational weight gain
Miremberg et al., 2018 (31)	Israel	60	60	Mobile phone	Usual care	To birth	Cesarean section, Emergency cesarean, Composite neonatal complication
Munda et al., 2023 (32)	Slovenia	53	52	Telemedicine device and video conferencing system	Usual care	To birth	Cesarean section, Composite neonatal complication, 2-h-OGTT, Gestational weight gain
Pérez-Ferre et al., 2010 (44)	Spain	49	48	Mobile phone	Usual care	To birth	Cesarean section, Gestational weight gain
Ping Yang et al., 2018 (50)	China	57	50	smartphones/WeChat	Usual care	Not Available	Cesarean section, 2-h-OGTT
Rasekaba et al., 2018 (49)	Australia	61	34	Internet	Usual care	To birth	Cesarean section, Emergency cesarean
Su et al., 2021 (52)	China	56	56	Internet	Usual care	6 months	Cesarean section
Sung et al., 2019 (34)	South Korea	11	10	Mobile phone	Usual care	To birth	Cesarean section, Gestational weight gain
Sun ying et al., 2021 (53)	China	40	40	Mobile phone	Usual care	To birth	Cesarean section
Cetinkaya et al., 2022	Turkey	23	22	Smartphone	Usual care	14 weeks	2-h-OGTT
Yew et al., 2021 (30)	Singapore	170	170	Telemedicine device and telephone	Usual care	To birth	Cesarean section, Emergency cesarean, Composite neonatal complication, Gestational weight gain

### 3.4 Risk of bias

**Figure 2:**
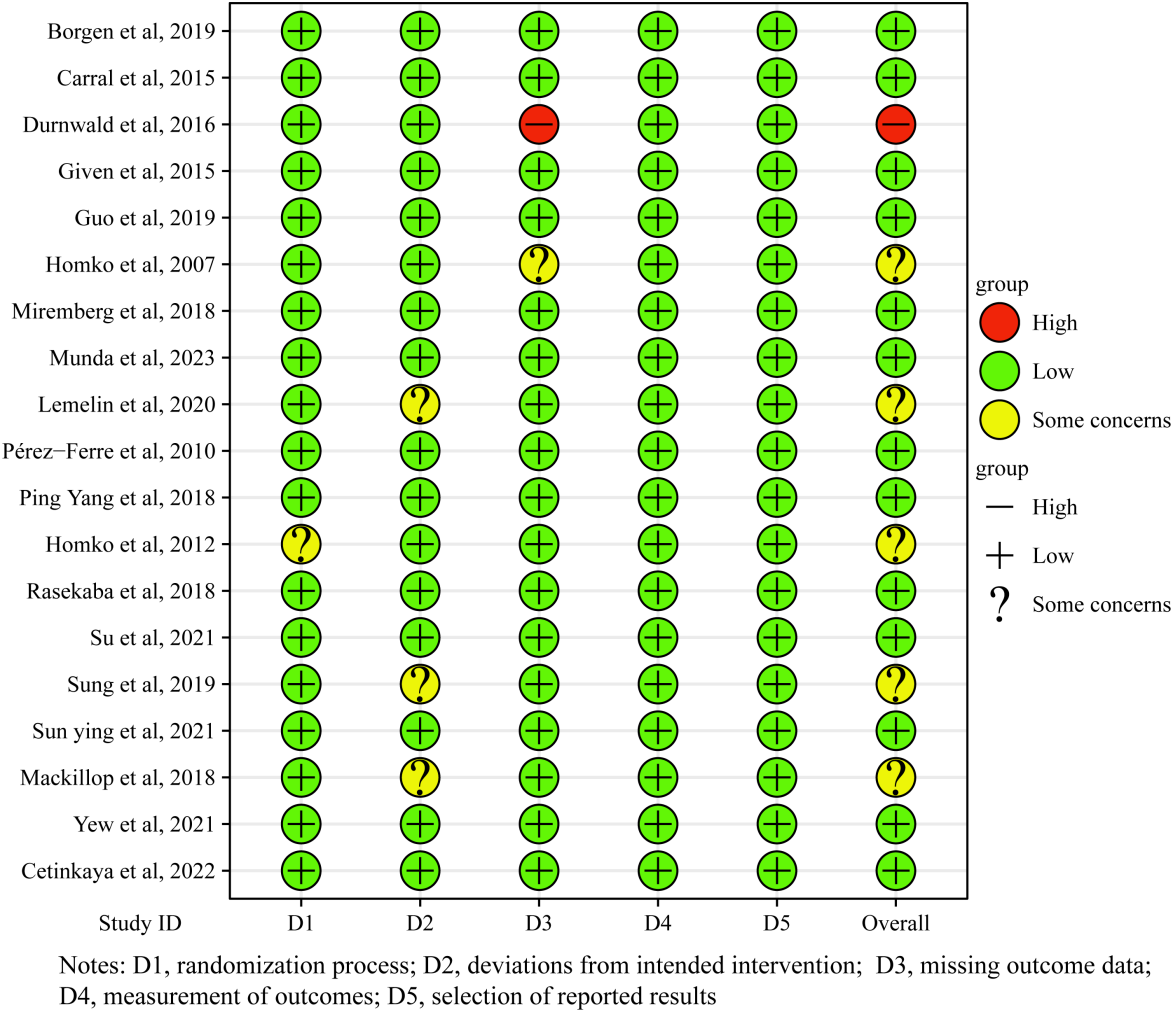
Risk of bias summary: Review authors' judgments about the risk of bias item for each included study.

As shown in **Figure 2**, among the 19 included studies, 13 were rated as having an overall low risk of bias, 5 studies were assessed to have some concerns regarding the risk of bias (34, 43, 45, 48, 51), and 1 study was rated as having a high risk of bias (35). Regarding the randomization process, except for one study that was evaluated as having some concerns (45), the remaining 18 studies reported adequate randomization procedures. They were considered to have a low risk of bias. Regarding deviations from intended interventions, 16 studies were rated as having a low risk of bias, while 3 studies were assessed to have some concerns (34, 48, 51). Regarding missing outcome data, 17 studies were judged as having a low risk of bias, one study raised some concerns (43), and another was rated as having a high risk of bias (35). In the domains of outcome measurement and selection of reported results, all 19 studies were evaluated as having a low risk of bias.

### 3.5 Outcomes from included studies

A systematic review and meta-analysis of 19 eligible randomized controlled trials was conducted to evaluate the impact of mHealth interventions on maternal and infant outcomes in pregnant women with GDM. The results demonstrated that mHealth interventions significantly reduced the incidence of cesarean section and emergency cesarean delivery among women with GDM, as well as significantly lowering the risk of composite neonatal complications. Additionally, mHealth interventions markedly improved the 2-hour postprandial blood glucose levels in GDM patients. Furthermore, a significant trend toward reduced gestational weight gain was observed in the mHealth intervention group. Detailed statistical results are presented as follows:

#### 3.5.1 Caesarean section

**Figure 3:**
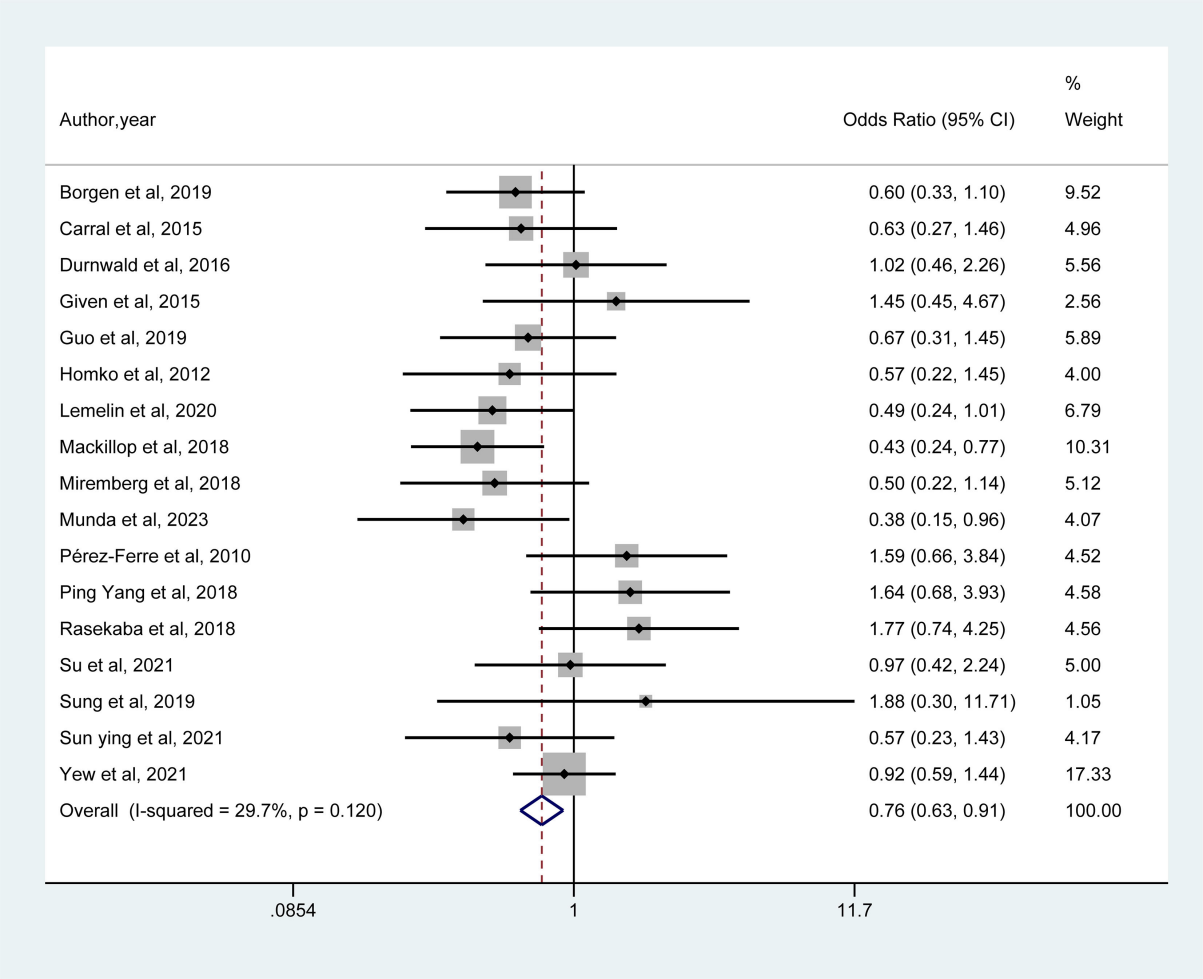
Forest plot for the efficacy of mHealth interventions on caesarean section.

As shown in **Figure 3**, a meta-analysis of 17 studies indicated that mHealth interventions significantly reduced the rate of caesarean section in women with gestational diabetes mellitus (GDM) compared with usual care (OR = 0.76, 95% CI = 0.63–0.91, I² = 29.7%).

#### 3.5.2 Emergency caesarean section

**Figure 4:**
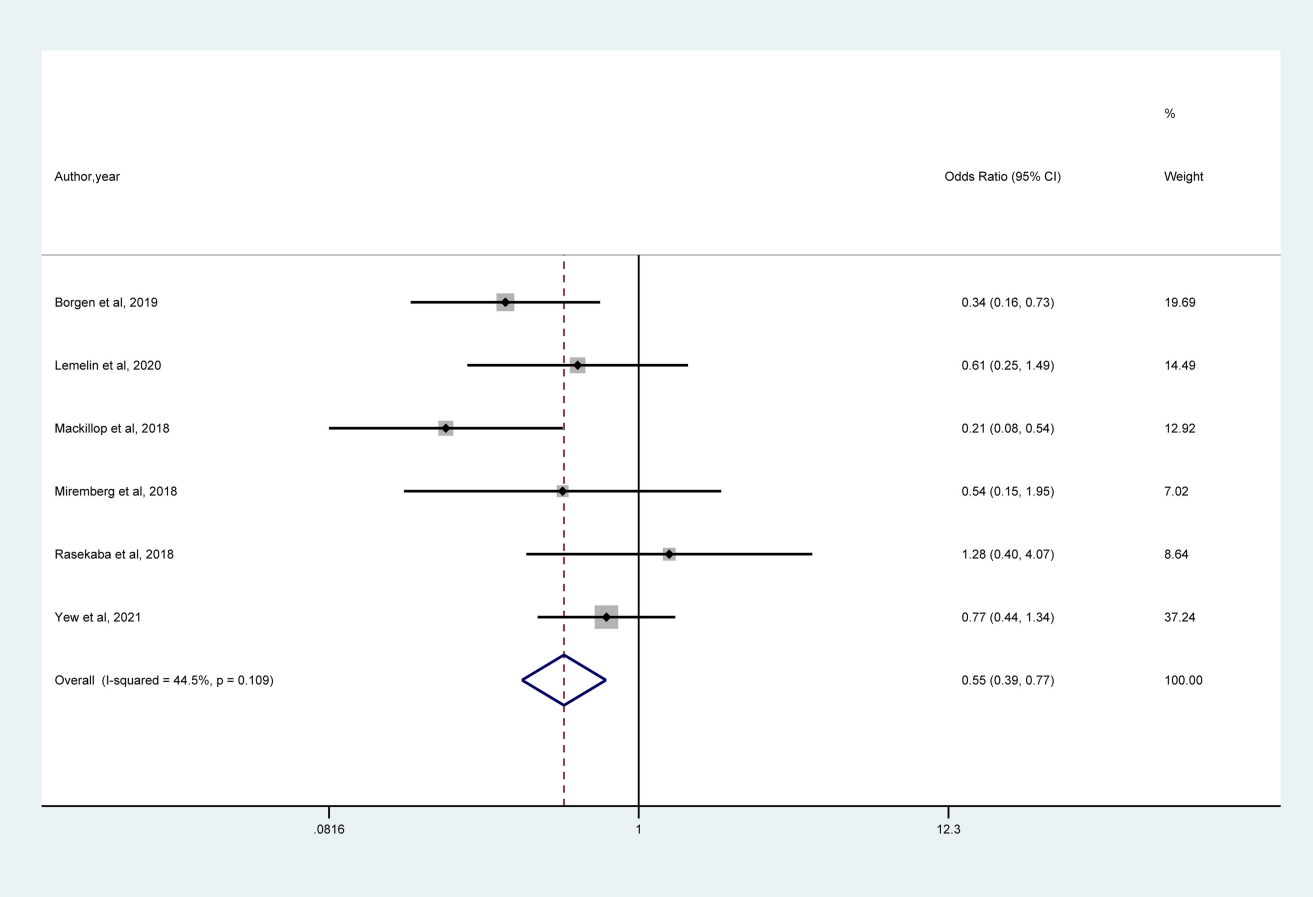
Forest plot for the efficacy of mHealth interventions on emergency cesarean.

As shown in **Figure 4**, a meta-analysis of 6 studies demonstrated that mHealth interventions significantly reduced the risk of emergency caesarean section in women with GDM compared with usual care (OR = 0.55, 95% CI = 0.39–0.77, I² = 44.5%).

#### 3.5.3 Composite neonatal complications

**Figure 5:**
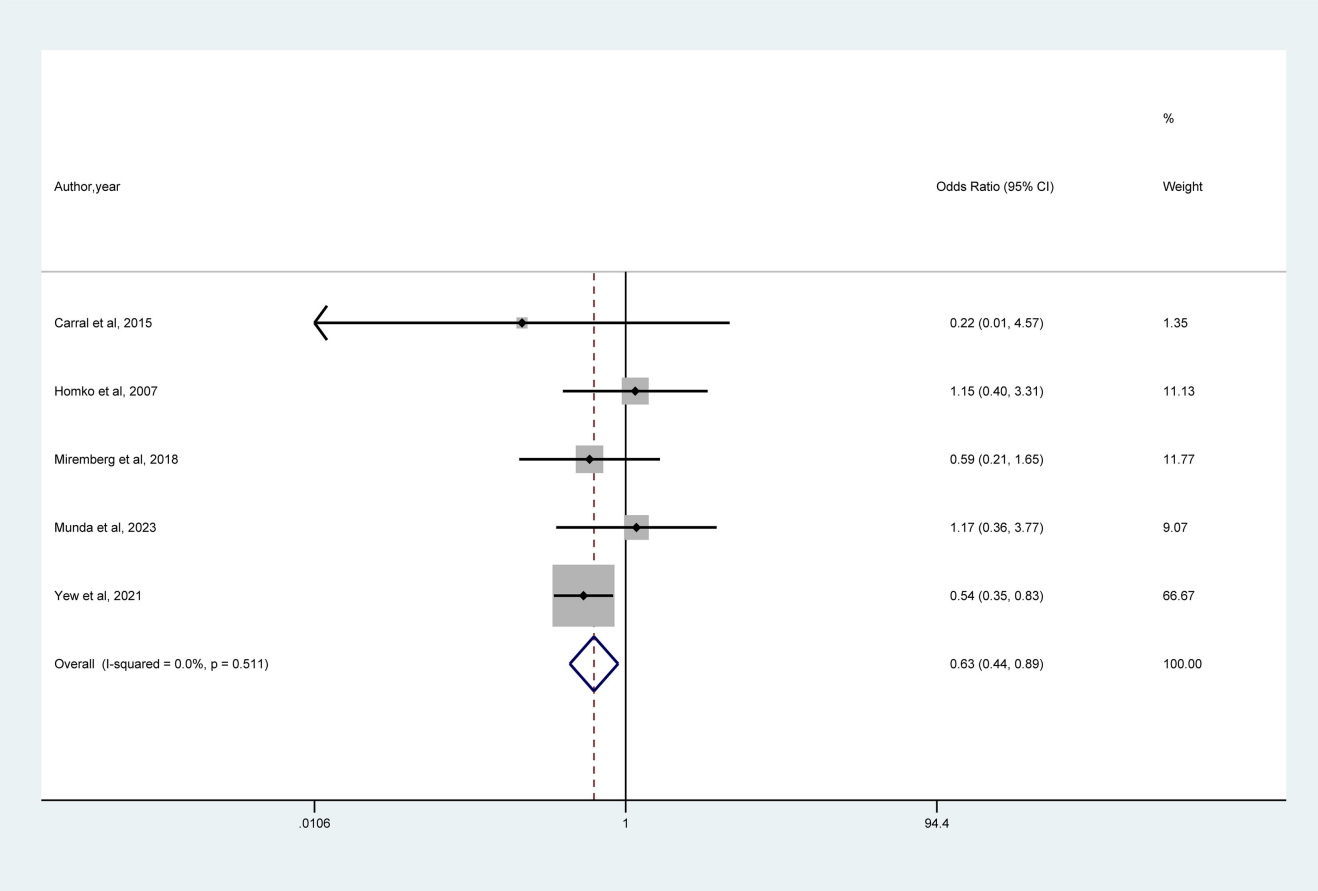
Forest plot for the efficacy of mHealth interventions on composite neonatal complication.

A meta-analysis of 5 studies (**Figure 5**) revealed that mHealth interventions significantly reduced the risk of composite neonatal complications (OR = 0.63, 95% CI = 0.44–0.89, I² = 0%).

#### 3.5.4 2-Hour postprandial blood glucose

**Figure 6:**
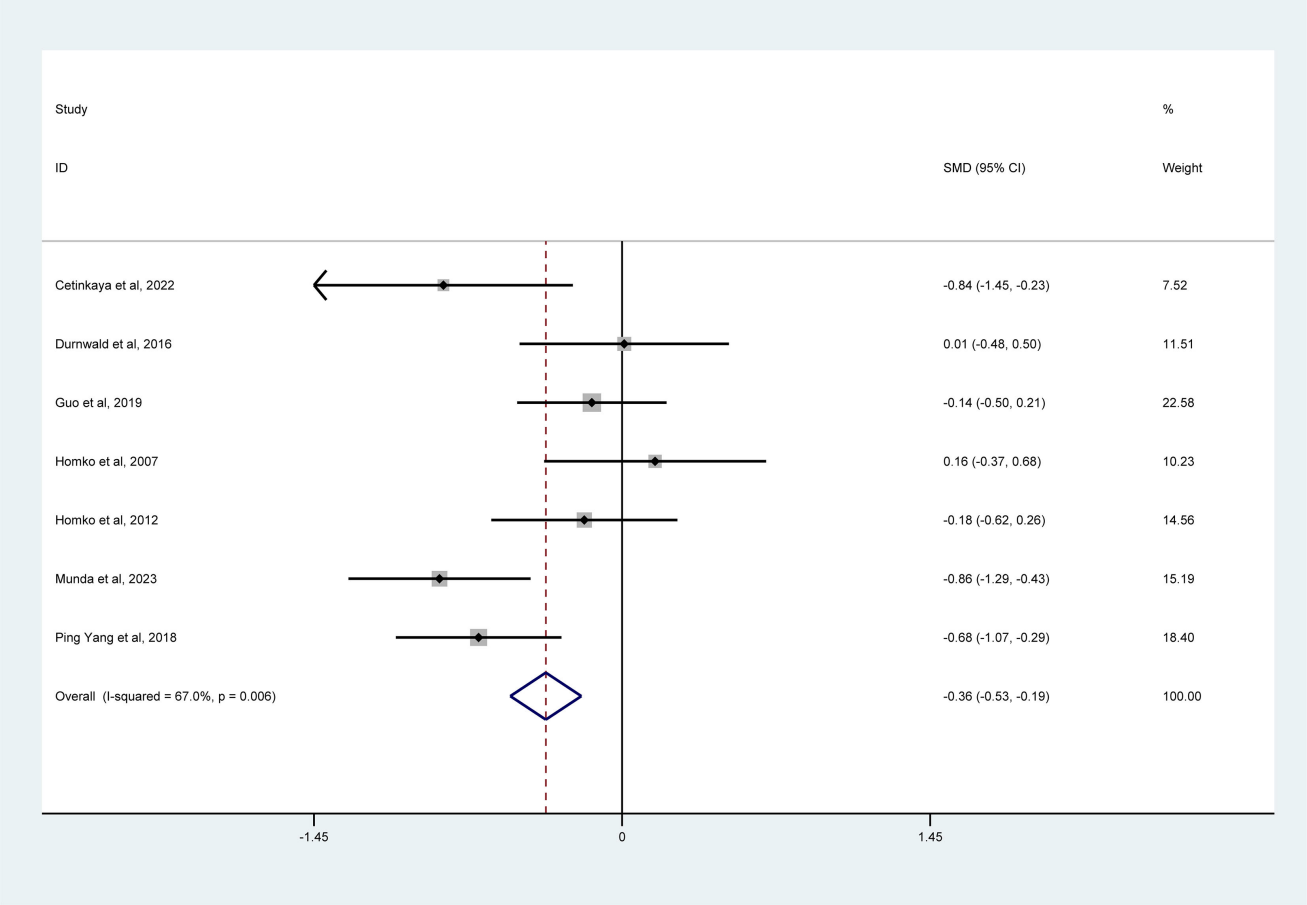
Forest plot for the efficacy of mHealth interventions on 2-hour postprandial blood glucose.

As shown in **Figure 6**, a meta-analysis of 7 studies indicated that mHealth interventions significantly improved 2-hour postprandial blood glucose levels in women with GDM (SMD = –0.36, 95% CI = –0.53 to –0.19, I² = 67%).

#### 3.5.5 Gestational weight gain

**Figure 7:**
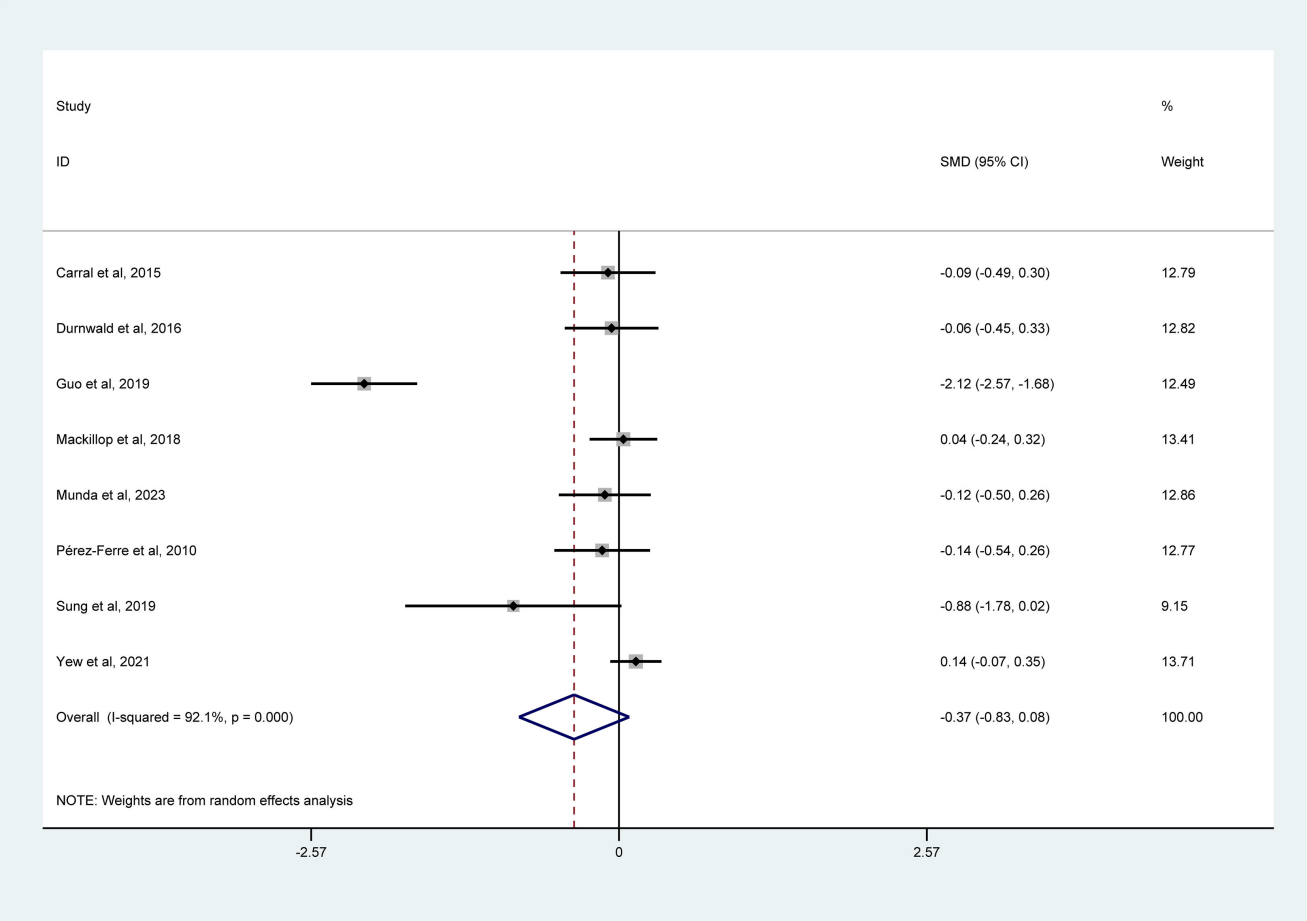
Forest plot for the efficacy of mHealth interventions on gestational weight gain.

A meta-analysis of 8 studies (**Figure 7**) suggested that gestational weight gain tended to be significantly lower in the mHealth intervention group than in the usual care group among women with GDM (SMD = –0.37, 95% CI = –0.83 to 0.08, I² = 92.1%).

### 3.6 Sensitivity analysis

The sensitivity analysis results are presented in **Supplementary Figures S1–S5**. The pooled effect estimates remained robust after the sequential exclusion of each study.

### 3.7 Publication bias

Both Begg’s test and Egger’s test (P > 0.05) indicated no significant publication bias in the results of this meta-analysis (**Supplementary Table S2**).

## 4 Discussion

GDM is a common metabolic disorder during pregnancy, and its global occurrence rate continues to rise (4, 5). GDM not only increases the risk of adverse pregnancy outcomes such as gestational hypertension and cesarean delivery (55) but may also lead to a series of complications, including macrosomia, neonatal hypoglycemia, and respiratory distress syndrome (56), posing serious threats to maternal and infant health. With the rapid development of digital health technologies, mobile health (mHealth) interventions have become a research focus in patient symptom management due to their advantages of convenience, real-time monitoring, and personalization (18). mHealth interventions offer multiple functions such as remote blood glucose monitoring, dietary and exercise guidance, health education delivery, and interactive communication between patients and healthcare providers (25, 26). However, findings from individual studies on the effectiveness of mHealth interventions in GDM management are not entirely consistent (29–35). Therefore, this systematic review and meta-analysis included 19 studies to evaluate the effects of mHealth interventions on cesarean section, emergency cesarean delivery, 2-hour postprandial blood glucose, gestational weight gain, and composite neonatal complications. The results indicate that mHealth interventions significantly improved maternal and neonatal outcomes in women with GDM. Specifically, mHealth interventions significantly reduced the rates of cesarean section and emergency cesarean delivery, improved 2-hour postprandial blood glucose levels, and resulted in significantly lower gestational weight gain in the intervention group. Regarding neonatal outcomes, mHealth interventions significantly reduced the risk of composite neonatal complications. Furthermore, Begg’s and Egger’s tests indicated no significant publication bias, and sensitivity analysis demonstrated that the findings were robust.

### 4.1 Multidimensional mechanisms of mHealth interventions on maternal and neonatal outcomes in GDM

In relation to caesarean section-related circumstances, mobile healthcare has played a multifaceted role. First, through wearable devices and mobile applications, mHealth enables real-time and continuous monitoring of maternal physiological indicators such as blood glucose, providing continuous glucose profiles to capture fluctuation patterns (13). If abnormal blood glucose levels are detected, the system automatically triggers alerts, facilitating immediate intervention (57). Healthcare providers can adjust treatment plans based on real-time data (58), effectively preventing macrosomia due to uncontrolled blood glucose and significantly reducing the associated risk of cesarean section (59). Second, mHealth applications can provide personalized dietary and exercise recommendations tailored to the individual condition of pregnant women (23). These behavior intervention plans, tailored to different risk levels, can enhance patient compliance and self-management capabilities (23). Additionally, pregnant women can use mHealth platforms to communicate with healthcare providers in real time and report data (60), thereby enhancing their confidence in self-management and enabling prompt resolution of clinical issues (60), thus avoiding emergency cesarean sections due to disease progression (61).

Regarding the improvement of neonatal outcomes, the real-time monitoring mechanism enables continuous tracking of maternal blood glucose levels, allowing for the timely detection and correction of abnormalities (13), thereby reducing fetal exposure to hyperglycemia (57) and preventing fetal metabolic disorders and related complications. Personalized support, which provides appropriate dietary and exercise plans based on the specific conditions of pregnant women (23), optimizes nutrient intake and energy expenditure (23), lowers the risk of intrauterine hyperglycemia (59), and reduces the occurrence rate of macrosomia, neonatal hypoglycemia, and respiratory distress syndrome (62). The continuous monitoring provided by mHealth offers data support, enabling more precise interventions and safeguarding neonatal health (57).

In terms of glycemic control, real-time monitoring enables pregnant women and healthcare providers to dynamically track blood glucose fluctuations via wearable devices (13). If abnormal postprandial glucose levels are detected, the system provides immediate alerts, prompting adjustments to dietary or physical activity regimens (63). Personalized support involves the development of individualized management plans based on the patient’s glucose profile and risk factors (23, 60). For instance, individuals with suboptimal glycemic control are advised to adopt high-fiber diets and engage in low-intensity exercise, which has been shown to improve the rate of achieving target glucose levels (59). Through mobile platforms, physicians and patients engage in interactive consultations, facilitating the timely resolution of patient concerns (60) and enhancing treatment adherence (23). Such professional guidance reinforces glycemic management and helps maintain glucose levels within a safe range (60).

Regarding gestational weight gain, mHealth interventions help pregnant women achieve appropriate weight control through personalized guidance on dietary and physical activity. These individualized plans are designed based on maternal physiological parameters to optimize energy balance (23) and are dynamically adjusted using real-time feedback on behavioral data (23). For example, when excessive weight gain is observed, the system or healthcare providers prompt the patient to increase physical activity or modify dietary intake, thereby mitigating risks associated with excessive gestational weight gain (12). Successful weight management has been associated with a reduced incidence of macrosomia and cesarean delivery (12).

In summary, mHealth interventions positively influence maternal and infant outcomes, including cesarean section rates, neonatal outcomes, glycemic control, and gestational weight gain among women with GDM through multidimensional mechanisms such as real-time monitoring, personalized support, and enhanced patient–provider interaction.

### 4.2 Comparison with the published systematic review and meta-analysis

Additionally, one study has evaluated the association between digital health interventions and maternal and neonatal outcomes (64). We compared the present study with the aforementioned research: Regarding literature search periods and databases, the study by Wang et al. (64) covered publications up to August 2024 and searched four databases (PubMed, Embase, Cochrane Library, and Web of Science). The current study, however, extended the search up to July 2025 and included five databases: PubMed, Web of Science, Scopus, Cochrane Library, and EMBASE. In terms of the number of included studies and study populations, Wang et al. (64) incorporated 42 relevant RCTs involving 148,866 pregnant women, whereas the present study included 19 RCTs involving 1,821 pregnant women with GDM. Regarding outcome measures, the study by Wang et al. (64) evaluated outcomes such as gestational weight gain, gestational hypertension or preeclampsia, gestational age at delivery, miscarriage, shoulder dystocia, preterm birth, infant birth weight, macrosomia, and neonatal hypoglycemia. In contrast, our study focused on five specific outcomes: caesarean section, emergency caesarean, composite neonatal complications, 2-hour postprandial blood glucose, and gestational weight gain. Furthermore, this study explored the potential mechanisms through which mHealth interventions may improve maternal and infant outcomes in GDM, thereby enriching both the research content and theoretical foundation. Finally, the conclusions drawn in this study were based on sensitivity analysis and publication bias detection, rendering them more robust and persuasive.

### 4.3 Limitations and strengths

#### 4.3.1 Limitations

Although this study employed rigorous methodological approaches for data analysis, several limitations should be considered when interpreting the results:

First, variations in the definition and diagnostic criteria for GDM across different studies may affect the accuracy of the findings. Second, due to the limited number of included studies and their clinical characteristics, subgroup analyses based on categories of mHealth interventions, specific intervention content, or duration of intervention were not conducted. Third, while this study focused on perinatal outcomes (such as cesarean delivery and neonatal complications), GDM has long-term implications. Most included studies only followed participants until delivery and did not assess the impact of mHealth interventions on long-term outcomes. Thus, whether such interventions can sustainably improve long-term maternal and infant health remains uncertain. Furthermore, given that the included studies span from 2007 to 2025, a considerable temporal gap exists during which digital technology has evolved substantially. As a result, it is challenging to clearly delineate the confounding effects of digital development on the intervention outcomes.

#### 4.3.2 Strengths

First, the study design enhances the strength of the evidence. Only randomized controlled trials were included, which significantly strengthens the evidence supporting the effect of mHealth interventions on maternal and infant outcomes in GDM. Second, the methodological design is rigorous, and the quality of evidence is high. This study strictly adhered to the PRISMA statement for reporting, registered the study protocol in PROSPERO, and involved two independent reviewers in literature screening, data extraction, and risk of bias assessment, thereby reducing subjective bias. A random-effects model was employed to address heterogeneity, and the robustness of the results was confirmed through sensitivity analysis using the leave-one-out method. Publication bias was ruled out via Begg’s test and Egger’s test, ensuring the reliability of the pooled effect estimates. Third, selecting outcome measures reflects both clinical relevance and practical utility. The study focused on outcomes of high clinical interest in GDM management, such as cesarean section, emergency cesarean section, composite neonatal complications, 2-hour postprandial blood glucose, and gestational weight gain. These indicators are central to perinatal quality assessment and are closely associated with short- and long-term maternal and infant health. The findings can directly inform clinical decision-making.

### 4.4 Implications for clinical practice

This study demonstrates that mHealth interventions can effectively optimize glycemic control and reduce the risks of emergency cesarean delivery and composite neonatal complications. These findings suggest that such interventions may compensate for the limitations of traditional healthcare models in follow-up management and real-time intervention, particularly offering a feasible solution for resource-limited settings. Healthcare providers should fully recognize the potential of mHealth in managing GDM. Through remote monitoring, intelligent reminders, and behavioral interventions, mHealth enhances patient compliance and self-management capabilities, thereby reducing hospitalization needs and long-term healthcare burdens. Furthermore, this study provides evidence-based support for updating clinical guidelines and informing policy-making, which may facilitate the integration of mHealth into standard GDM care and promote the advancement of personalized medicine and interdisciplinary collaboration. Future research should further explore AI-driven precision interventions and the long-term effects of mHealth on offspring health, thereby establishing a more solid theoretical and practical foundation for applying digital healthcare in the perinatal period.

## 5 Conclusion

This systematic review and meta-analysis incorporated 19 RCTs to comprehensively evaluate the effectiveness of mHealth interventions on maternal and infant outcomes in GDM. The results demonstrated that mHealth interventions can reduce the rates of cesarean section and emergency cesarean section, decrease the risk of neonatal composite complications, and improve 2-hour postprandial blood glucose levels in pregnant women with GDM. These findings highlight the potential value of mHealth interventions in GDM management, effectively supplementing conventional clinical care for GDM. Further rigorous, high-quality, and large-sample RCTs are warranted to validate these findings.

## Data Availability

The original contributions presented in the study are included in the article/**Supplementary Material**. Further inquiries can be directed to the corresponding author.
