# Supplementary material for: The impact of mobile health interventions on maternal-neonatal outcomes in women with gestational diabetes mellitus: a systematic review and meta-analysis
Source: Front Endocrinol (Lausanne). 2025 Dec 19;16:1707520. doi: 10.3389/fendo.2025.1707520 (PMC12757257; doi:10.3389/fendo.2025.1707520)
Supplement: Supplementary file 1 [file DataSheet1.docx]

The Impact of Mobile Health (mHealth) Interventions on Maternal-Neonatal Outcomes in Women with Gestational Diabetes Mellitus: A Systematic Review and Meta-Analysis

[1 Definition of Outcome Measures. 2](#_Toc22049)

[2 Table S1 Search strategy of each database. 3](#_Toc562)

[3 Table S2 Begg’s and Egger’s test values 6](#_Toc27825)

[4 Sensitivity analysis - Caesarean section - Figure S1 7](#_Toc17230)

[5 Sensitivity analysis - Emergency cesarean - Figure S2 8](#_Toc5791)

[6 Sensitivity analysis - Composite neonatal complication - Figure S3 9](#_Toc2785)

[7 Sensitivity analysis - 2-hour postprandial blood glucose - Figure S4 10](#_Toc18540)

[8 Sensitivity analysis - Gestational weight gain - Figure S5 11](#_Toc20995)

[9 Figure Legends 12](#_Toc31493)

# 1 Definition of Outcome Measures.

**Caesarean section:**

Surgical delivery of a fetus through incisions in the abdominal wall (laparotomy) and uterine wall (hysterotomy) . **PMID: 38812964**

**Emergency cesarean:**

Unplanned cesarean delivery performed due to immediate threat to maternal or fetal life (e.g., fetal distress, cord prolapse, or severe hemorrhage). **PMID: 40947089**

**2-hour postprandial blood glucose:**

Plasma glucose concentration measured 2 hours after the start of a meal, with thresholds for gestational diabetes mellitus (GDM) diagnosis typically >120 mg/dL (>6.7 mmol/L) . **PMID: 33998636**

**Gestational weight gain:**

Total weight gained during pregnancy from conception to delivery, categorized by pre-pregnancy BMI according to Institute of Medicine guidelines. **PMID: 40102908**

**Composite neonatal complication:**

A combined endpoint including neonatal death, respiratory distress syndrome, hypoxic-ischemic encephalopathy, intraventricular hemorrhage (grade >I), necrotizing enterocolitis, sepsis, or prolonged NICU admission (≥4 days). **PMID: 37265117**

# 2 Table S1 Search strategy of each database.

| **1. Pubmed** | | |
| --- | --- | --- |
| #1 | (((((((((((((((((((((((Phone[Title/Abstract]) OR (Mobile phone[Title/Abstract])) OR (smart phone[Title/Abstract])) OR (telephone[Title/Abstract])) OR (android[Title/Abstract])) OR (Ipad[Title/Abstract])) OR (Computer*[Title/Abstract])) OR (application[Title/Abstract])) OR (mobile health[Title/Abstract])) OR (web-based[Title/Abstract])) OR (internet based[Title/Abstract])) OR (online*[Title/Abstract])) OR (e-mail*[Title/Abstract])) OR (e-health*[Title/Abstract])) OR (eTherap*[Title/Abstract])) OR (telehealth[Title/Abstract])) OR (telemedicine[Title/Abstract])) OR (teletherap*[Title/Abstract])) OR (facebook[Title/Abstract])) OR (wechat[Title/Abstract])) OR (twitter[Title/Abstract])) OR (microblog[Title/Abstract])) OR (digital health*[Title/Abstract])) OR (e-care*[Title/Abstract]) | 2,057,722 |
| #2 | ((Diabetes, Gestational[Title/Abstract]) OR (Gestational Diabetes Mellitus[Title/Abstract])) OR (Diabetes, Pregnancy-Induced[Title/Abstract]) | 16,355 |
| #3 | #1 AND #2 | 770 |
| **2. Web of science** | | |
| #1 | Phone (Abstract) or Mobile phone (Abstract) or smart phone (Abstract) or telephone (Abstract) or android (Abstract) or Ipad (Abstract) or Computer* (Abstract) or application (Abstract) or mobile health (Abstract) or web-based (Abstract) or internet based (Abstract) or online* (Abstract) or e-mail* (Abstract) or e-health* (Abstract) or eTherap* (Abstract) or telehealth (Abstract) or telemedicine (Abstract) or teletherap* (Abstract) or facebook (Abstract) or wechat (Abstract) or twitter (Abstract) or microblog (Abstract) or digital health* (Abstract) or e-care* (Abstract) and Preprint Citation Index (Exclude – Database) | 8,589,633 |
| #2 | "Diabetes, Gestational" (Abstract) or "Gestational Diabetes Mellitus" (Abstract) or "Diabetes, Pregnancy-Induced" (Abstract) and Preprint Citation Index (Exclude – Database) | 16,825 |
| #3 | #1 AND #2 | 951 |
| **3. Scopus** | | |
| #1 | ( TITLE-ABS-KEY ( Phone ) OR TITLE-ABS-KEY ( "Mobile phone" ) OR TITLE-ABS-KEY ( "smart phone" ) OR TITLE-ABS-KEY ( telephone ) OR TITLE-ABS-KEY ( android ) OR TITLE-ABS-KEY ( Ipad ) OR TITLE-ABS-KEY ( Computer* ) OR TITLE-ABS-KEY ( application ) OR TITLE-ABS-KEY ( "mobile health" ) OR TITLE-ABS-KEY ( web-based ) OR TITLE-ABS-KEY ( "internet based" ) OR TITLE-ABS-KEY ( online* ) OR TITLE-ABS-KEY ( e-mail* ) OR TITLE-ABS-KEY ( e-health* ) OR TITLE-ABS-KEY ( eTherap* ) OR TITLE-ABS-KEY ( telehealth ) OR TITLE-ABS-KEY ( telemedicine ) OR TITLE-ABS-KEY ( teletherap* ) OR TITLE-ABS-KEY ( facebook ) OR TITLE-ABS-KEY ( wechat ) OR TITLE-ABS-KEY ( twitter ) OR TITLE-ABS-KEY ( microblog ) OR TITLE-ABS-KEY ( "digital health*" ) OR TITLE-ABS-KEY ( e-care* ) ) | 15,487,811 |
| #2 | ( TITLE-ABS-KEY ( "Diabetes, Gestational" ) OR TITLE-ABS-KEY ( "Gestational Diabetes Mellitus" ) OR TITLE-ABS-KEY ( "Diabetes, Pregnancy-Induced" ) ) | 25,182 |
| #3 | #1 AND #2 | 1,409 |
| **4. Embase** | | |
| #1 | phone OR 'mobile phone':ti,ab,kw OR 'smart phone':ti,ab,kw OR telephone:ti,ab,kw OR android:ti,ab,kw OR ipad:ti,ab,kw OR computer*:ti,ab,kw OR application:ti,ab,kw OR 'mobile health':ti,ab,kw OR 'web based':ti,ab,kw OR 'internet based':ti,ab,kw OR online*:ti,ab,kw OR 'e mail*':ti,ab,kw OR 'e health*':ti,ab,kw OR etherap*:ti,ab,kw OR telehealth:ti,ab,kw OR telemedicine:ti,ab,kw OR teletherap*:ti,ab,kw OR facebook:ti,ab,kw OR wechat:ti,ab,kw OR twitter:ti,ab,kw OR microblog:ti,ab,kw OR 'digital health*':ti,ab,kw OR 'e care*':ti,ab,kw | 2,635,498 |
| #2 | 'diabetes, gestational'/exp OR 'diabetes, gestational' OR 'gestational diabetes mellitus':ti,ab,kw OR 'diabetes, pregnancy-induced':ti,ab,kw | 62,898 |
| #3 | #1 AND #2 | 2,687 |
| **5. Cochrane Library** | | |
| #1 | (Phone):ti,ab,kw OR ("Mobile phone"):ti,ab,kw OR ("smart phone"):ti,ab,kw OR (telephone):ti,ab,kw OR (android):ti,ab,kw OR (Ipad):ti,ab,kw OR (Computer*):ti,ab,kw OR (application):ti,ab,kw OR ("mobile health"):ti,ab,kw OR (web-based):ti,ab,kw OR ("internet based"):ti,ab,kw OR (online*):ti,ab,kw OR (e-mail*):ti,ab,kw OR (e-health*):ti,ab,kw OR (eTherap*):ti,ab,kw OR (telehealth):ti,ab,kw OR (telemedicine):ti,ab,kw OR (teletherap*):ti,ab,kw OR (facebook):ti,ab,kw OR (wechat):ti,ab,kw OR (twitter):ti,ab,kw OR (microblog):ti,ab,kw OR (digital NEXT health*):ti,ab,kw OR (e-care*):ti,ab,kw | 226,647 |
| #2 | ("Diabetes, Gestational"):ti,ab,kw OR ("Gestational Diabetes Mellitus"):ti,ab,kw OR ("Diabetes, Pregnancy-Induced"):ti,ab,kw | 2821 |
| #3 | #1 AND #2 | 462 |

# 3 Table S2 Begg’s and Egger’s test values

| **Clinical symptom** | **T, P (Egger's Test)** | **Z, P (Begg's Test)** |
| --- | --- | --- |
| Caesarean section | 1.03, 0.318 | 1.19, 0.232 |
| Emergency cesarean | -0.31, 0.771 | 0.38, 0.707 |
| Composite neonatal complication | 0.58, 0.605 | 0.24, 0.806 |
| 2-hour blood glucose | -0.01, 0.990 | 0.60, 0.548 |
| Gestational weight gain | -1.56, 0.170 | 2.06, 0.09 |

# 4 Sensitivity analysis - Caesarean section - Figure S1


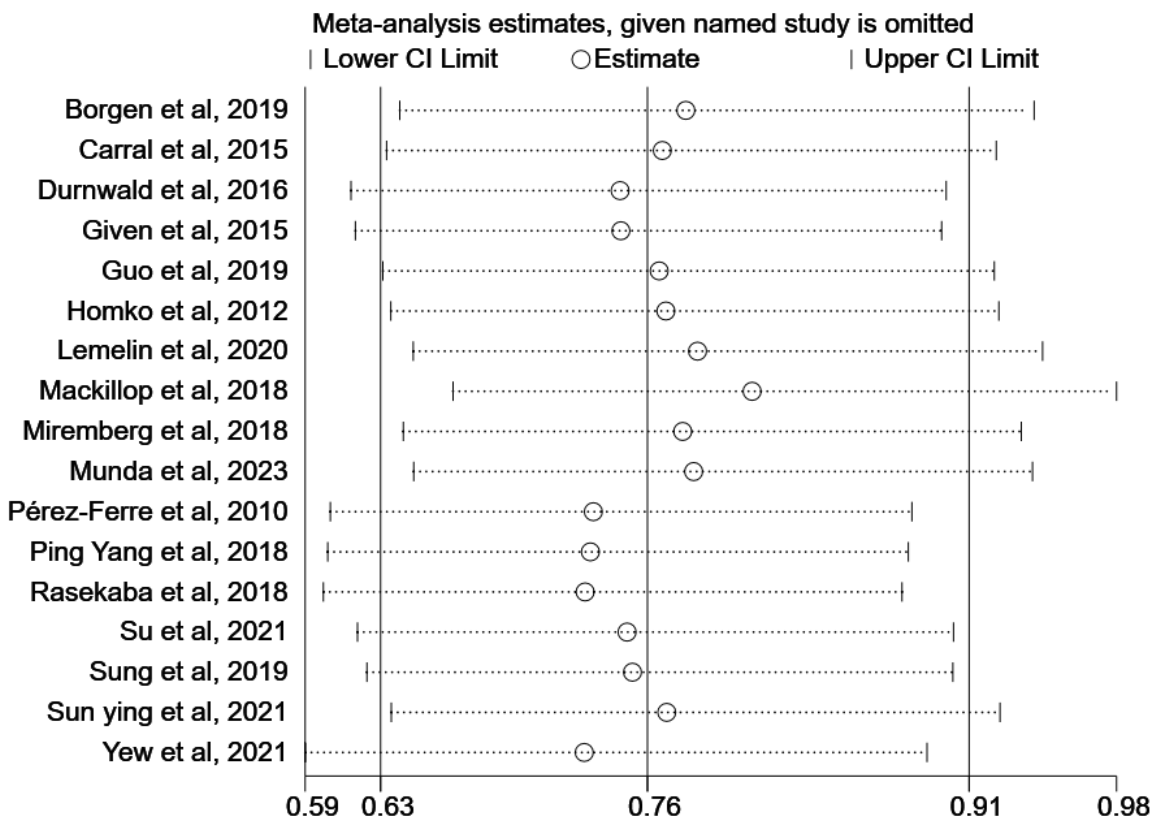


# 5 Sensitivity analysis - Emergency cesarean - Figure S2


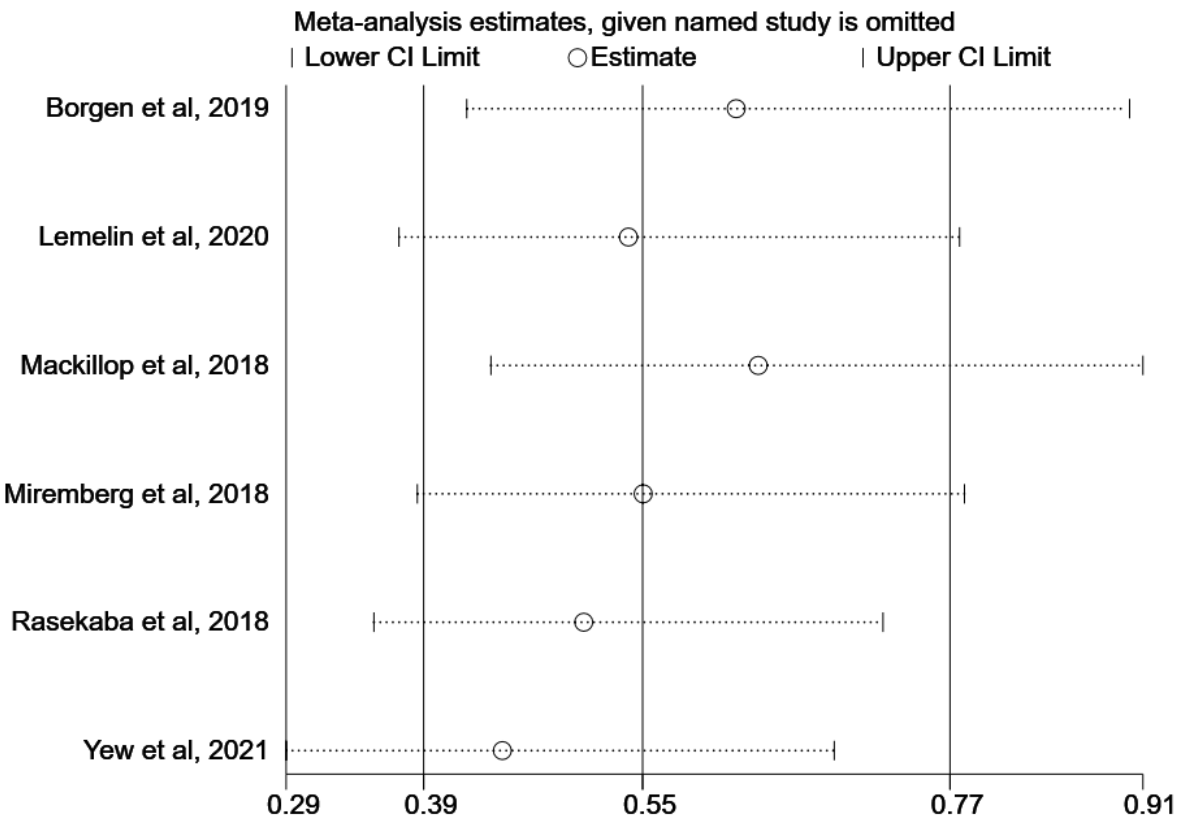


# 6 Sensitivity analysis - Composite neonatal complication - Figure S3


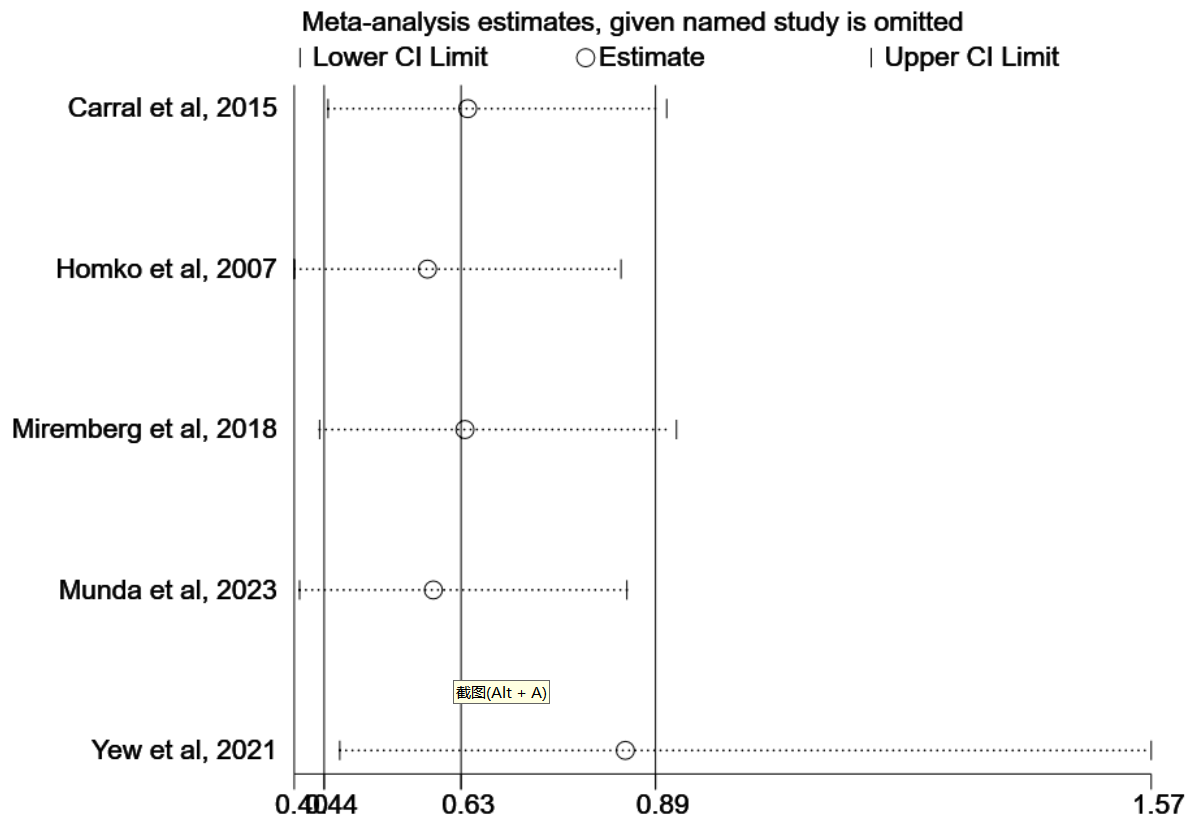


# 7 Sensitivity analysis - 2-hour postprandial blood glucose - Figure S4


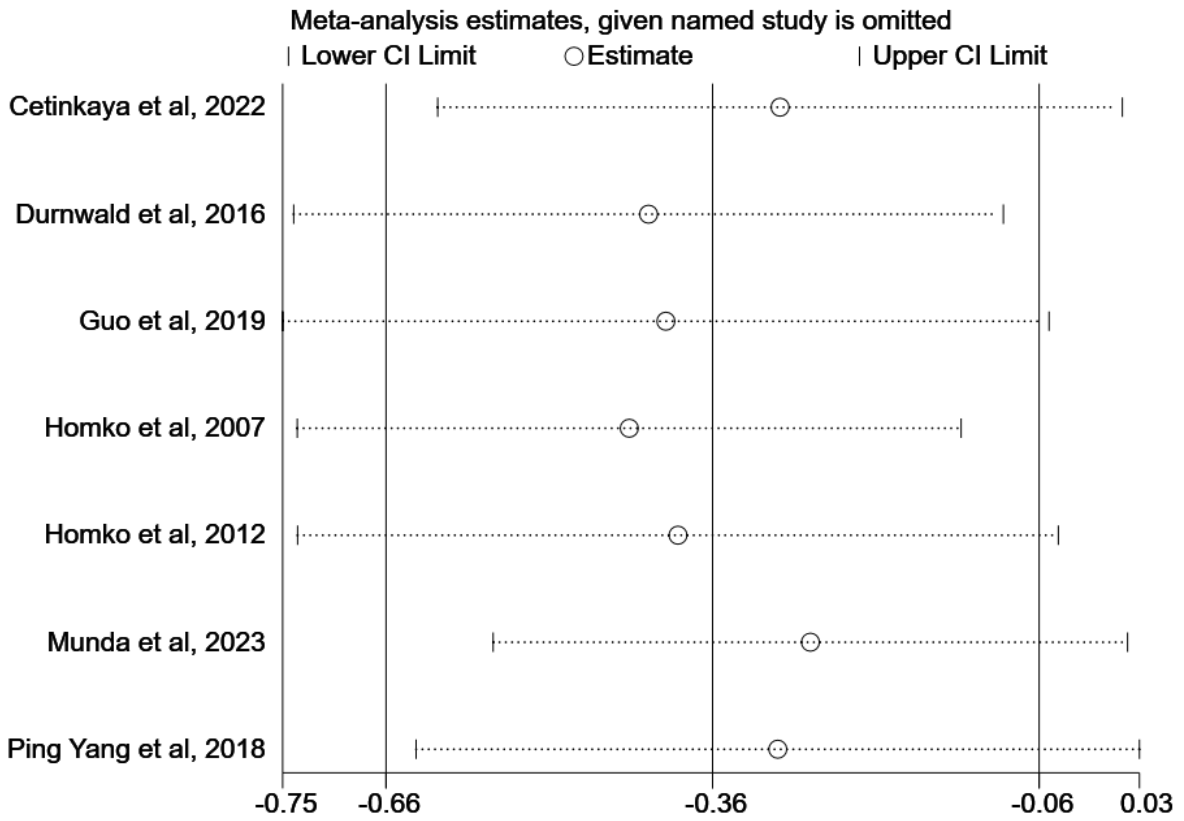


# 8 Sensitivity analysis - Gestational weight gain - Figure S5


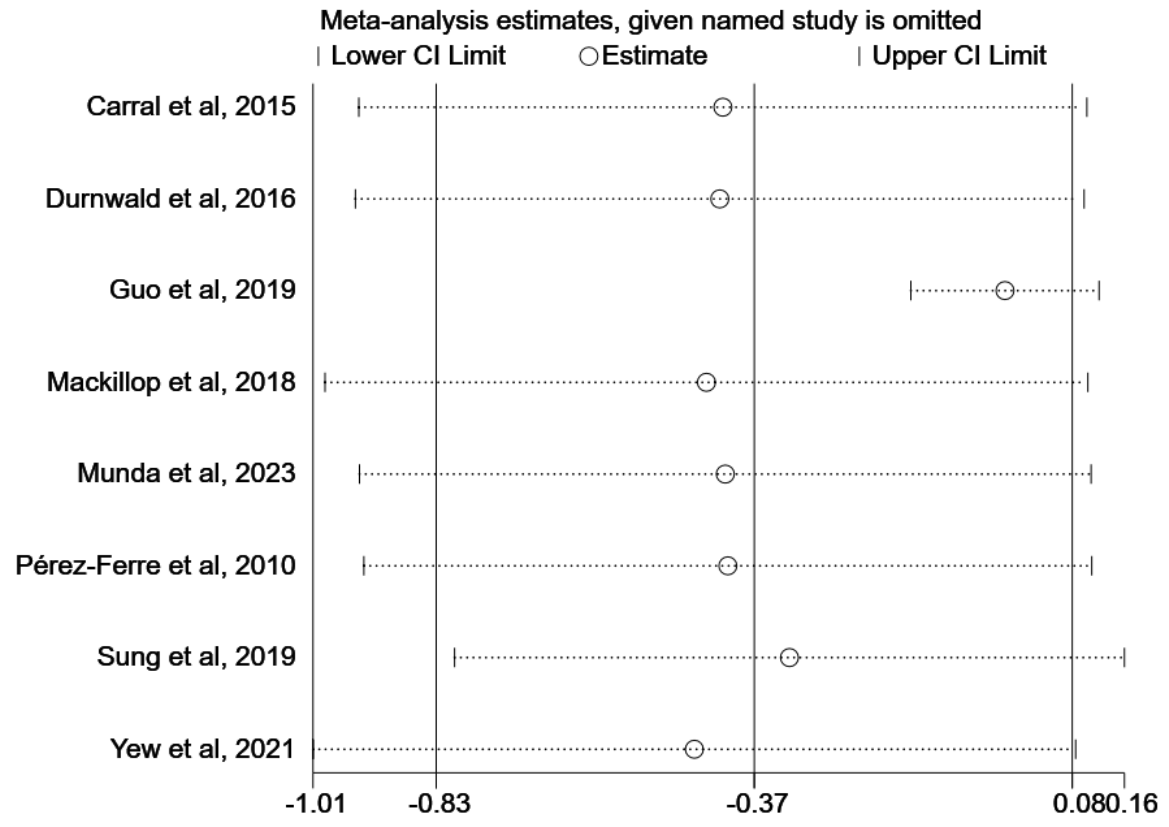


# 9 Figure Legends

Figure 1: PRISMA flow chart for study selection.

Figure 2: Risk of bias summary: Review authors' judgments about the risk of bias item for each included study.

Figure 3: Forest plot for the efficacy of mHealth interventions on caesarean section.

Figure 4: Forest plot for the efficacy of mHealth interventions on emergency cesarean.

Figure 5: Forest plot for the efficacy of mHealth interventions on composite neonatal complication.

Figure 6: Forest plot for the efficacy of mHealth interventions on 2-hour postprandial blood glucose.

Figure 7: Forest plot for the efficacy of mHealth interventions on gestational weight gain.
